# Evaluation of free radical scavenging capacity of methoxy containing-hybrids of thiosemicarbazone-triazole and their influence on glucose transport

**DOI:** 10.1186/s40360-018-0266-6

**Published:** 2018-12-06

**Authors:** Ademola O. Ayeleso, Jitcy S. Joseph, Oluwafemi O. Oguntibeju, Emmanuel Mukwevho

**Affiliations:** 1 Department of Biochemistry, North-West University, Mmabatho, 2735 South Africa; 2 Department of Biochemistry, Adeleke University, Ede, Osun State P.M.B. 250 Nigeria; 3 Department of Life & Consumer Sciences, University of South Africa, Florida Campus, Johannesburg, 1709 South Africa; 4 Oxidative Stress Research Centre, Department of Biomedical Sciences, Cape Peninsula University of Technology, Bellville, 7535 South Africa

**Keywords:** Hybrid compound, Thiosemicarbazone, Triazole, Free radical, Diabetes

## Abstract

**Background:**

Diabetes mellitus is a metabolic disease in which the body is unable to produce insulin or respond to insulin production, consequently leading to abnormal metabolism of carbohydrates, lipids and proteins causing elevation of glucose in the blood. Oxidative stress, an imbalance between the production of free radicals and body antioxidant system has been implicated in the pathogenesis of diabetes. Free radicals attack important macromolecules leading to cell damage. Antioxidants are intimately involved in the prevention of damage caused by free radicals.

**Methods:**

The anti-diabetic effects of hybrid compounds (**2a-h**) of thiosemicarbazone and triazole containing methoxy groups at C (4) positions were tested against genes involved in glucose metabolism (*Glut-4*, *Mef2a* and *Nrf-1*) using quantitative real time PCR (qPCR). Free radical scavenging capacity (FRAP, TEAC, DPPH and ORAC) of the hybrids was also carried out by using established antioxidant capacity assays.

**Results:**

From the results, hybrid compounds **2b** and **2h** showed more pronounced effects in up-regulating diabetes associated genes which are important in the up-regulation of glucose uptake. All the hybrid compounds also showed free radical scavenging abilities.

**Conclusion:**

In conclusion, hybrid compounds (**2b** and **2h**) can be useful as potential drugs for the management of diabetes mellitus.

## Background

Thiosemicarbazones are biologically active compounds which are obtained by the condensation of thiosemicarbazide or a substituted thiosemicarbazide with a suitable aldehyde or ketone. Thiosemicarbazones derivatives have been reported to have antidiabetic, antiviral, anticancer, antibacterial, antifungal, and antimalarial effects [1–8]. 1,2,3-Triazole is a heterocyclic compound which belong to a class of azole. A triazole is a five-membered aromatic ring which contains at least one nitrogen atom and another heteroatom such as nitrogen, oxygen, or sulphur in the ring. Triazole derivatives are known to have antifungal, antibacterial, anticancer, antimalaria, anti-inflammatory activities [9–14]. Derivatives of triazole have also been reported to protect pancreatic β cells against endoplasmmic reticulum stress-mediated dysfunction and death [15]. Hybrid compounds of thiosemicarbozone and triazole have been evaluated for their biological activities against malaria, obesity and diabetes [16–18].

A free radical is any molecular species that is capable of independent existence, possessing an unpaired electron in an atomic orbital [19]. Examples of free radicals include superoxide (O2•−), hydroxyl (OH•), peroxyl (RO2•), hydroperoxyl (HO2•), alkoxyl (RO•), peroxyl (ROO•), nitric oxide (NO•), nitrogen dioxide (NO2•) and lipid peroxyl (LOO•) [20]. Antioxidants are molecules which can safely interact with free radicals and terminate the chain reaction before vital molecules are damaged, inhibiting the oxidation of susceptible biomolecules such as proteins, lipids and DNA [21] thus, playing a role in the prevention of oxidative damage to the body. Antioxidants are known to exhibit antioxidant activity by donation of hydrogen atoms or single-electron transfer to a radical [22].

**Fig. 1:**
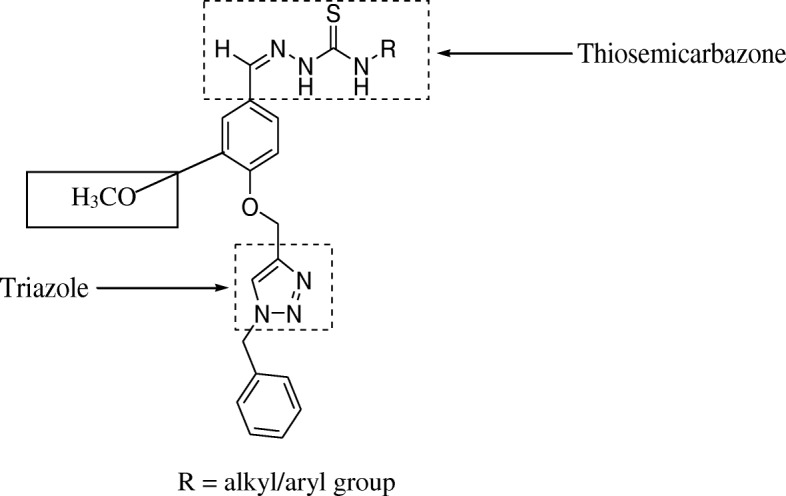
General chemical structure of the thiosemicarbazone- triazole hybrid compound with methoxy aromatic linker

Type 2 diabetes mellitus is a disease where the body either produces little insulin / ceases to produce insulin, or becomes progressively resistant to its action [23]. Upon stimulation by insulin, GLUT4 is a glucose transporter that is responsible for the uptake of glucose molecules into muscle cells and adipose tissue and it is reported to be indirectly regulated by NRF-1 through transcription of the gene myocyte enhancer factor 2 (MEF2) [18, 24, 25]. MEF2 is a transcription factor that binds to the promoter of the GLUT4 gene which in turn, regulates its transcription and expression [25, 26]. Experimental analysis on the antidiabetic and antioxidant potentials had earlier been done on hybrids compounds from thiosemcarbazone and triazole [18]. These compounds were further improved through the addition of a functional group (aromatic ring containing methoxy) at C (4) position (Fig. 1). The aim of this study was therefore to investigate antioxidant potentials i.e. free radical scavenging ability of the improved synthesized hybrid compounds of thiosemcarbazone and triazole as well as their influence on the expression of some genes associated with type 2 diabetes.

## Methods

### Synthesis of hybrid compounds

The synthesis of the hybrid compounds (**2b-h)** were synthesized in a similar fashion as published by [17, 18] except for the addition of an electron donating group (methoxy substituent to C(4) position of the aromatic ring joining thiosemicarbazone and triazole..

### Collection of cell lines

3T3-L1 adipocytes cell lines used in this study were collected from Prof E.O. Ojuka at the Department of Human Biology, University of Cape Town, South Africa. The cell lines were originally from American Type Culture Collection (ATCC) through Prof J.O. Holloszy at the Washington University School of Medicine, Missouri, USA.

### Cell culture and treatments

3T3-L1 adipocytes cell lines were cultured using Dulbecco’s modified Eagles medium (DMEM) (GIBCO, USA) supplemented with 10% Foetal calf serum (BioWest, France) and 1% penicillin/streptomycin/fungizone (GIBCO, USA) at 37 °C with 5% CO2 and 95% humidity. The maintenance of the cells was done in continuous passage by trypsinization of subconfluent cultures with Trypsin/Versene (Highveld, RSA). Differentiation was induced by introduction of medium containing 2% Foetal calf serum and 2% penicillin/ streptomycin/fungizone when pre-adipocytes were 80% confluent. Cells were kept in this medium for 5 days until adipocytes were well formed. Differentiated 3T3-L1 pre-adipocytes were treated with 5 μL of compounds (10 mg/mL) 2b-h or 100 nM insulin or Metformin for 4 h.

### Quantitative real-time PCR

Total RNA was isolated and purified from the treated cells using QIAzol lysis reagent (QIAGEN Sciences, USA) and RNA clean and Concentrator-25 (Inqaba Biotech, SA). Double stranded cDNA was synthesized from 3 μg of the total RNA using Superscript Reverse Transcriptase III (Invitrogen, USA). Real time quantitative PCR was done in triplicate using Rotor gene-3000 quantitative real-time PCR machine using Sensi Mix SYBR No-ROX One-Step Kit (Bioline, UK). The primers used were mouse *Glut4* gene (Forward primer- 5’ GCA GCG AGT GAC TGG AAC A 3′; Reverse primer- 5’CCA GCC ACG TTG CAT TGT AG 3′), *Nrf-1* gene (Forward primer- 5′ AAA CAC AAA CTC AGG CCA CC 3′; Reverse primer-5’ CCA TCA GCC ACA GCA GAG CA 3′) and *Mef2a* gene (Forward primer-5′ GTG TAC TCA GCA ATG CCG AC 3′; and Reverse primer-5’ AAC CCT GAG ATA ACT GCC CTC 3′). The amplification occurred in a 3-step cycle: denaturation at 95 °C for 5 s, annealing at 60 °C for 10 s, and extension at 72 °C for 15 s. Relative mRNA expression was normalised to mouse Actin reference gene (Forward primer- 5′ GAG ACC TTC AAC ACC CCA GCC 3′; Reverse primer- 5′ GGA GAG CAT AGC CCT CGT AG 3′) and calculated according to relative standard method.

### ABTS radical scavenging activity

This assay was carried out using the principle of 2,2- azino-bis (3-ethylbenzothiazoline-6 sulphonic acid) (ABTS) radical scavenging activity according to [27]. ABTS+ solution was prepared and left overnight before use by mixing ABTS salt (8 mM) with potassium persulfate (3 mM) and then storing the solution in the dark until the assay could be performed and ABTS^+^ solution was then diluted with distilled water. The sample (25 μl) was mixed with 1 ml ABTS^+^ solution (300 μl) and left for 30 min at room temperature. The sample was read at a wavelength of 734 nm. Trolox was used as the standard and results were expressed as μmol TE/g sample. All determinations were done in triplicates.

### Ferric reducing antioxidant power (FRAP) assay

The FRAP assay was done using the method described by [28]. The sample (10 μl) was mixed with 300 μl FRAP reagent (a mixture of acetate buffer (pH 3.6), tripyridyl triazine (TPTZ) and FeCl_3_ ·6H_2_O). After incubation at room temperature for 30 min, the samples were read at a wavelength of 593 nm. Ascorbic acid was used as the standard and the results were expressed as μmol AAE/g sample. All determinations were done in triplicates.

### DPPH free radical scavenging activity

DPPH free radical scavenging activity of the sample was carried out according to a modified method of [29]. The sample (10 μl) was reacted with DPPH solution (190 μl) and the absorbance was determined after 30 min at a wavelength of 517 nm. Free radical scavenging activity of the samples was expressed according to the equation below:

Percent (%) inhibition of DPPH activity

$$ \frac{A^o-A}{A^o}\times 100, $$

Where *A*° is the absorbance of DPPH∙ in solution without an antioxidant and *A* is the absorbance of DPPH∙ in the presence of an antioxidant.

### Oxygen radical absorbance capacity (ORAC) assay

The ORAC assay was carried out according to the method of [30] using a fluorescence plate reader (Thermo Fisher Scientific, Waltham, Mass., USA). The reaction consisted of 12 μl of diluted aqueous plant extracts and 138ul of fluorescein (14 μM), which was used as a target for free radical attack. The reaction was initiated by the addition of 50 μl AAPH (768 μM) and the fluorescence (emission 538 nm, excitation 485 nm) recorded every 1 min for 2 h in triplicates. Trolox was used as the standard and results expressed as μmol TE/g sample.

### Statistical analysis

Results are presented as means ± SD. Statistical analysis was performed by one-way ANOVA followed by the Tukey’s post hoc test. The level of significance was accepted at *P* < 0.05. All statistical analyses were performed using GraphPad InStat 3 software.

## Results

### Free radical scavenging abilities of the hybrids

**Fig. 2:**
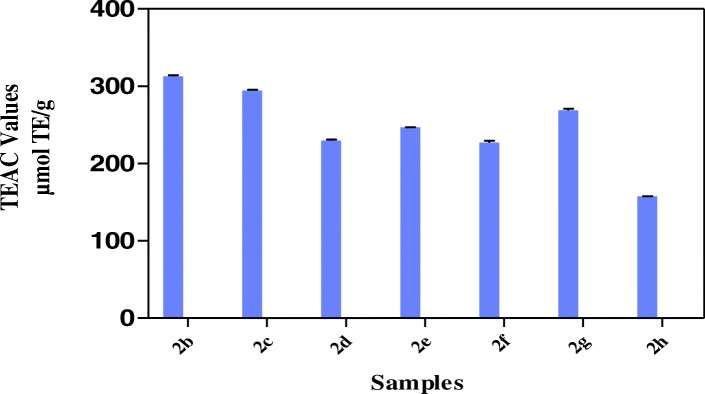
ABTS radical scavenging activity of the hybrid compounds

**Fig. 3:**
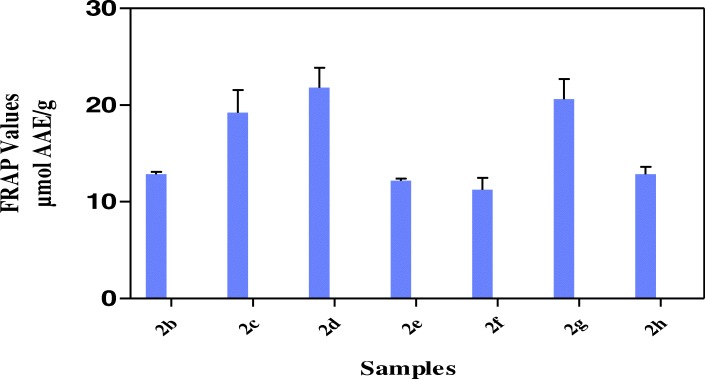
Ferric reducing antioxidant power of the hybrid compounds

**Fig. 4:**
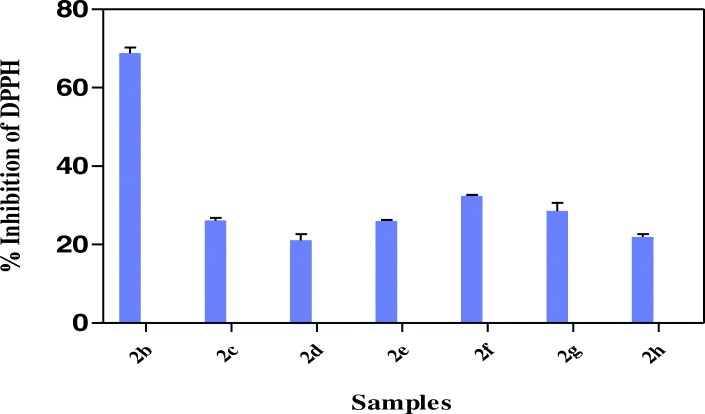
DPPH radical scavenging activity of the hybrid compounds

**Fig. 5:**
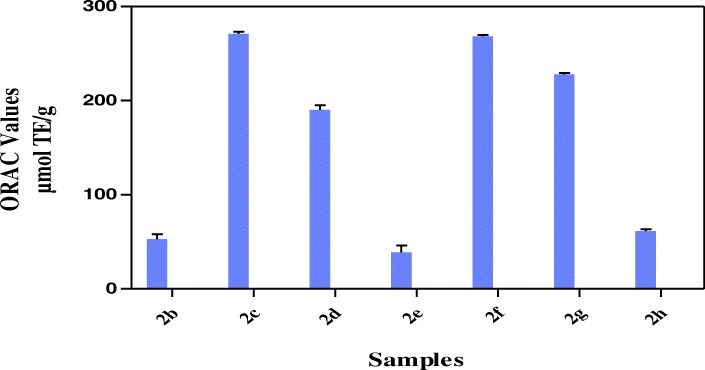
Oxygen radical absorbance capacity of the hybrid compounds

The results in Fig. 2 showed that all the hybrid compounds possess hydrogen or electron donating abilities through ABTS assay in relation trolox as standard with hybrid compound **2b** showing the highest TEAC value of 312.9 ± 1.41 μmol TE/g. Other hybrid compounds had TEAC values of 294.6 ± 1.10 μmol TE/g (**2c**), 229.8 ± 1.21 μmol TE/g (**2d**), 246.7 ± 0.40 μmol TE/g (**2e**), 227.1 ± 2.55 μmol TE/g (**2f**), 268.9 ± 2.09 μmol TE/g (**2f**) and 157.4 ± 0.64 μmol TE/g (**2h**). The results in Fig. 3 showed the strength of the reducing power of the hybrid compounds with **2c, 2d** and **2g** having FRAP values of 19.2 ± 2.37 μmol AAE/g, 21.81 ± 2.06 μmol AAE/g, 20.62 2.09 μmol AAE/g respectively, followed by **2b, 2e, 2f** and **2h** with FRAP values of 12.9 ± 0.25 μmol AAE/g, 12.2 ± 0.21 μmol AAE/g, 11.25 ± 1.23 μmol AAE/g and 12.9 ± 0.76 μmol AAE/g respectively. Among the hybrid compounds, **2b** had the most pronounced inhibition of DPPH (68.9% ± 1.40) while others have 26.1% ± 0.69 (**2c**), 21.1% ± 1.59 (**2d**), 26.0% ± 0.29 (**2e**), 32.4 ± 0.37 (**2f**), 28.5% ± 2.12 (**2g**), 22.0% ± 0.69 (**2 h**) inhibition of DPPH (Fig. 4). All the hybrid compounds showed inhibition of peroxyl radical with **2c, 2d, 2f** and **2g** having ORAC values of 270.9 ± 2.12 μmol TE/g., 190.2 ± 4.92 μmol TE/g., 268.3 ± 1.29 μmol TE/g and 227.9 ± 1.59 μmol TE/g respectively followed by **2b, 2e,** and **2h** with ORAC values of 53.0 ± 5.11 μmol TE/g, 38.84 ± 7.31 μmol TE/g and 61.4 ± 2.00 μmol TE/g respectively (Fig. 5).

### *Glut-4* gene expression in response to treatment with the hybrids

**Fig. 6:**
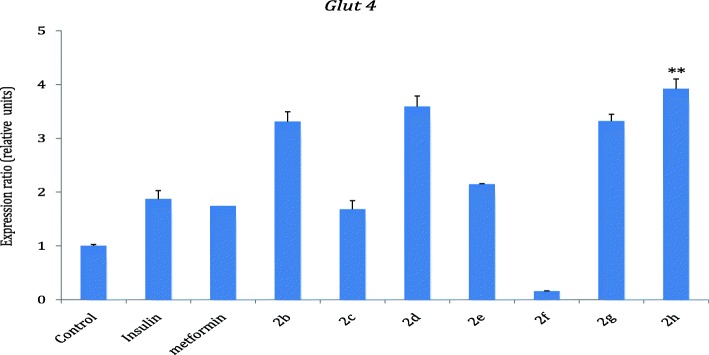
*Glut-4* expression in response to treatment with hybrids 2b-h. Results are presented as means ± SD. Level of significance was accepted at *P* < 0.05. The *P* value, control vs 2 h *P* < 0.01 (**), Insulin vs 2 h *P* < 0.01 (**)

The results in Fig. 6 showed that all the hybrid compounds except **2f** stimulated the expression of *glut-4* better than control. Hybrid compounds **2b**, **2d**, **2e**, **2g** and **2h** showed expression of *glut-4* better than the standard drugs, insulin and metformin when compared with the control. Hybrid compound **2h** showed a more pronounced increase (3.9 fold of the control) and was followed by hybrid compounds **2b, 2d and 2g** with 3.3, 3.6 and 3.3 folds increase respectively when compared with the control.

### *Mef2a* gene expression in response to treatment with the hybrids

**Fig. 7:**
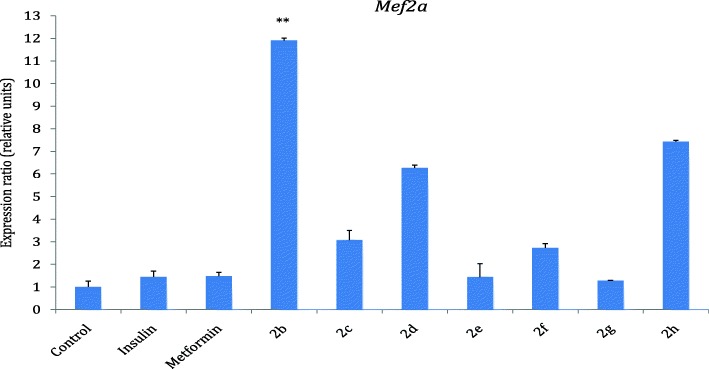
*Mef2a* expression in response to treatment with hybrids 2b-h. Results are presented as means ± SD. Level of significance was accepted at *P* < 0.05. The *P* value, control vs 2b *P* < 0.01 (**), Insulin vs 2b *P* < 0.01 (**)

The results in Fig. 7 showed that the hybrid compounds **2b**, **2c**, **2d**, **2f** and **2h** expressed *Mef2a* better than standard drugs, insulin and metformin relative to the control. The effect of **2b** was almost 12 fold increase in the expression of *Mef2a* followed by hybrid compounds **2d** and **2h** which exhibited 6.3 and 7.4 folds increase, respectively relative to the control.

### *Nrf-1* gene expression in response to treatment with the hybrids

**Fig. 8:**
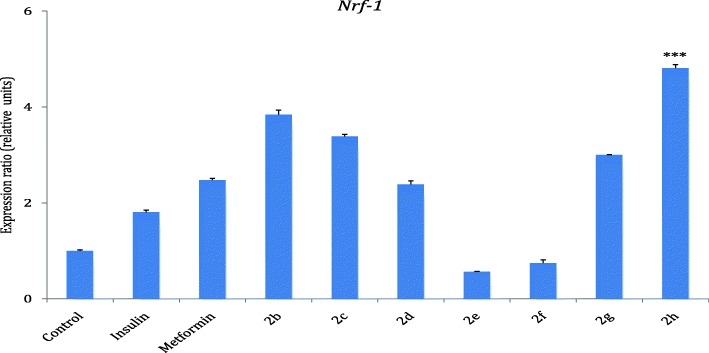
*Nrf-1* expression in response to treatment with hybrids 2b-h. Results are presented as means ±SD. Level of significance was accepted at *P* < 0.05. The *P* value, control vs 2 h *P* < 0.001 (***), Insulin vs 2 h *P* < 0.001 (***)

The result in Fig. 8 showed that all the hybrid compounds except **2e** and **2f** stimulated the expression on *Nrf-1* gene in relative to the control. Hybrid compounds **2b**, **2c**, **2 g** and **2h** expressed *Nrf-1* better than standard drugs, insulin and metformin. Hybrid compound **2h** showed a more pronounced increase in expression (4.8 fold of the control) while **2b, 2c and 2g** showed 3.8, 3.4 and 3 folds increase in the expression of *Nrf-1* gene relative to the control.

## Discussion

Antioxidants prevent cell and tissue damage as they act as free radical scavengers, neutralizing the electrical charges on free radicals and thereby hinders them from accepting electrons from other molecules [31, 32]. Elevated levels of free radical molecules result in oxidative stress in cells, leading to the destruction of vital macromolecules including DNA, lipids, and proteins [33]. Oxidative stress, an imbalance between the production of free radicals and the ability of the body antioxidant system to fight back, has been implicated in the pathogenesis of many chronic diseases including diabetes mellitus. Antioxidants alleviate oxidative stress, the adverse effects of free radical [34] and reportedly help in slowing down aging process and fight diseases such as diabetes mellitus, hypertension and cancer [32, 35].

Hybrid compounds, **2b, 2d** and **2c** are non-polar long alkyl chains of the amine moiety while non-polar short alkyl chains of the amine moiety are hybrids, 2a, 2e and 2f (Table 1). Hybrids compounds, **2g and 2h** are aryl containing amine groups (Table 1). In this study, the biological activities of hybrids (**2b-h**), containing electron donating group (methoxy group) at the aromatic linker of both thiosemicarbazone and triazole at C4 position were investigated for their free radical scavenging abilities and expression of genes involved in glucose uptake. ABTS assay is an excellent means for determining the antioxidant activity of hydrogen-donating and chain-breaking antioxidants [36]. It involves electron transfer process and is based on discolorations of ABTS by antioxidant compounds thus reflecting the amount of ABTS radicals that are scavenged within a fixed time period in relation to that of trolox [37]. In this study, ABTS scavenging ability reported as trolox equivalence antioxidant capacity (TEAC) revealed that the hybrid compound **2b** had the highest value which was followed by **2c** while **2h** had the lowest (Fig. 2).

**Table 1:**
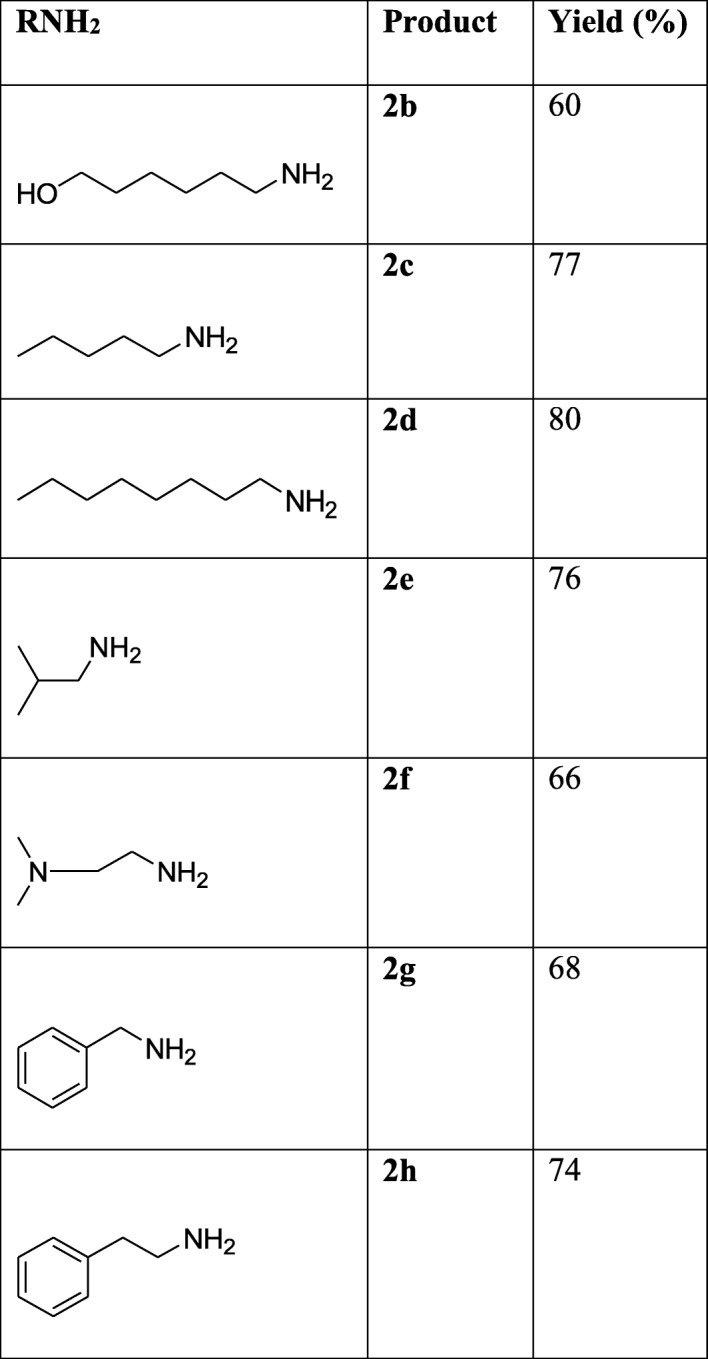
Synthesized thiosemicarbazone-triazole hybrid compounds (R = alkyl/aryl group)

The FRAP assay is reproducible and linearly related to the molar concentration of the antioxidant [38] and the reducing capacity of a compound could be used as an important indicator of its possible antioxidant activity [39]. In reducing power assay, antioxidants act as electron donor which reduces the Fe^3+^ complex to its Fe^2+^ and the reducing power is indicated by higher absorbance values [34]. In this study, assay of reducing activity was based on the reduction of ferric to the ferrous form in the presence of reductants (antioxidants) in the tested hybrid compounds and measuring the greater absorbance of blue colour solution at 700 nm showed greater reducing power. The values for the reducing power (from Fe^3+^ to Fe^2+^) of the hybrid compounds was reported as ascorbic acid equivalents (Fig. 3) with **2d** having highest FRAP value, followed by **2g** while **2f** was the lowest. The results indicated that all the hybrid compounds had reducing properties, thus revealed their antioxidative potentials.

In this study, DPPH inhibition value shows the antioxidant ability of the hybrid compounds by accepting an electron or hydrogen radical to become a stable diamagnetic molecule. DPPH assay has been widely used as quick, reliable, and reproducible parameter for showing in vitro antioxidant activity reducing the violet colour to yellow coloured product in the presence of antioxidant [34]. DPPH• is a stable radical showing a maximum absorbance at 515 nm and method is based on the reduction of DPPH• in alcoholic solution in the presence of a hydrogen-donating antioxidant due to the formation of the non-radical form DPPH-H in the reaction [40]. The study showed that the highest DPPH inhibition value was **2c** and followed by **2f** while the **2d** was the lowest (Fig. 4). The results confirmed that the hybrids were able to reduce the stable radical DPPH to the yellow-colored diphenylpicrylhydrazone suggesting their scavenging potentials by its proton donating ability.

The ORAC assay uses 2,2-azobis(2-amidinopropane) dihydrochloride (AAPH) for free radical generation and measures the antioxidant inhibition of peroxyl-radical-induced oxidations which shows the radical chain breaking antioxidant activity by H atom transfer [41]. All the hybrid compounds showed ORAC values **2c** having the highest with **2f** as the next while the lowest value was **2e** (Fig. 5). The results also confirm the antioxidant potency of the hybrid compounds.

GLUT4 is a glucose transporter that is responsible for the uptake of glucose molecules into muscle cells and adipose tissue upon stimulation by insulin [25]. The expression of *Glut4* in relation to the test compounds was compared with the control. As shown in Fig. 6, all the hybrid compounds except **2f** showed more expression of *Glut4* than the control with hybrid compounds **2b, 2d, 2e, 2g,** and **2h** stimulating *Glut4* expression more than insulin and metformin.

Furthermore, *Mef2a* gene expression was investigated on compound **2b-h**. (Fig. 7). MEF2 is a transcription factor that binds to the promoter of the GLUT4 gene thereby regulating its transcription and expression. Ramachandran et al [24] has also shown that the transcription of the gene myocyte enhancer factor 2 (MEF2) is regulated by NRF-1. *Mef2a* was better expressed by **2b**, **2c**, **2d**, **2f**, **2h** than the standard drugs, insulin and metformin relative to the control.

In addition, Hybrids **2b-h** were also tested for their effect on the expression of *Nrf-1* gene (Fig. 8). The test results showed that all the hybrids except **2e** and **2f** stimulated the expression of *Nrf-1* gene relative to the control (Fig. 8), with hybrids **2b, 2c, 2g,** and **2h** expressing *Nrf-1* better than insulin and metformin relative to the control. Overall, hybrid **2b** with a non-polar short alkyl chain of the amine moiety and **2h**, an aryl containing amine group were consistently up-regulating *Glut-4*, *Mef2a* and *Nrf-1*.

## Conclusion

The study showed the abilities of the hybrid compounds in trapping free radicals which are present in biological systems from a wide variety of sources. This study also revealed that hybrids **2b** and **2h** had consistent stimulatory effects on glucose uptake as shown on the expression of *Glut-4, Mef2a* and *Nrf-1.* These compounds might be important in the up- regulation of glucose uptake. It can be deduced from the results that the hybrids could help in the scavenging of free radicals and thus inhibit the oxidative mechanisms that could lead to diabetic complications. Hence, this study shows that these compounds can be useful as therapeutic agents in the treatment of diabetes mellitus.

## Abbreviations

AAE: Ascorbic Acid Equivalent
ABTS: 2,2- azino-bis (3-ethylbenzothiazoline-6 sulphonic acid)
ATCC: American Type Culture Collection
DPPH: 2,2-diphenyl-1-picrylhydrazyl
FRAP: Ferric Reducing Antioxidant Power
*Glut-4*: Glucose transporter 4
*Mef2a*: Myocyte enhancing factor 2A
Nrf-1: Nuclear respiratory factor-1
ORAC: Oxygen Radical Absorbance Capacity
qPCR: Quantitative real time PCR
TE: Trolox Equivalent
